# Macrophage migration inhibitory factor (MIF) and its homolog D-dopachrome tautomerase (D-DT) are significant promotors of UVB- but not chemically induced non-melanoma skin cancer

**DOI:** 10.1038/s41598-023-38748-9

**Published:** 2023-07-18

**Authors:** Sebastian Huth, Laura Huth, Ruth Heise, Yvonne Marquardt, Linda Lopopolo, Marta Piecychna, Peter Boor, Günter Fingerle-Rowson, Aphrodite Kapurniotu, Amir S. Yazdi, Richard Bucala, Jürgen Bernhagen, Jens Malte Baron

**Affiliations:** 1 Department of Dermatology and Allergology, Medical Faculty, RWTH Aachen University, Pauwelsstrasse 30, 52074 Aachen, Germany; 2 Department of Medicine, Yale School of Medicine, New Haven, CT USA; 3 Institute of Pathology and Department of Nephrology and Immunology, Medical Faculty, RWTH Aachen University, Aachen, Germany; 4 Department I of Internal Medicine, Center of Integrated Oncology Köln Bonn, University Hospital of Cologne, Cologne, Germany; 5 Division of Peptide Biochemistry, School of Life Sciences, Technical University of Munich (TUM), Freising, Germany; 6 Division of Vascular Biology, Institute for Stroke and Dementia Research (ISD), Ludwig-Maximilians-University (LMU), Munich, Germany

**Keywords:** Skin cancer, Cancer models, Oncology, Cancer

## Abstract

Non-melanoma skin cancer (NMSC) is the most common cancer in Caucasians worldwide. We investigated the pathophysiological role of MIF and its homolog D-DT in UVB- and chemically induced NMSC using *Mif*^*−/−*^, *D-dt*^*−/−*^ and *Mif*^*−/−*^*/D-dt*^*−/−*^ mice on a hairless SKH1 background. Knockout of both cytokines showed similar attenuating effects on inflammation after acute UVB irradiation and tumor formation during chronic UVB irradiation, without additive protective effects noted in double knockout mice, indicating that both cytokines activate a similar signaling threshold. In contrast, genetic deletion of *Mif* and *D-dt* had no major effects on chemically induced skin tumors. To get insight into the contributing mechanisms, we used an in vitro 3D skin model with incorporated macrophages. Application of recombinant MIF and D-DT led to an accumulation of macrophages within the epidermal part that could be reversed by selective inhibitors of MIF and D-DT pathways. In summary, our data indicate that MIF and D-DT contribute to the development and progression of UVB- but not chemically induced NMSC, a role at least partially accounted by effects of both cytokines on epidermal macrophage accumulation. These data highlight that MIF and D-DT are both potential therapeutic targets for the prevention of photocarcinogenesis but not chemical carcinogenesis.

## Introduction

Non-melanoma skin cancer (NMSC) is the most common cancer in Caucasians with increasing incidence worldwide as a result of chronic exposure to environmental factors such as ultraviolet B (UVB) radiation and chemicals^1,2^. Chronic skin exposure to these exogenous noxae stimulates the production of cytokines with pivotal roles in inflammatory reactions and skin tumor development^3^.

Macrophage migration inhibitory factor (MIF) is a pleiotropic cytokine that is unique in its structure and biological activities, also combining the characteristics of a chemokine and growth factor^4^. MIF has been shown to play a key role in innate and adaptive immune responses^5–8^. For its interaction with target cells MIF binds to the surface receptors CD74/CD44, CXCR2, CXCR4 and/or CXCR7 (a.k.a. ACKR3), in a context- and cell type-dependent manner^9–12^. Increasing evidence indicates that MIF is an important link between chronic inflammation and tumorigenesis^13^. While the exact mechanisms by which MIF elicits its inflammatory effects are not fully understood, MIF has been shown to inhibit p53-dependent growth arrest and apoptosis to sustain activation responses^13,14^. MIF also has been described to be a pro-tumorigenic factor in various cancers that promotes angiogenesis, increases cell proliferation, and modulates tumor immunity^15–17^. The expression of MIF is increased in most solid and hematological malignancies and is often considered a negative prognostic indicator^8,18^. In this context, MIF may be a promising target for therapies^19,20^.

In the skin, high expression levels of MIF were found in the basal layer of the epidermis and cutaneous appendages^10,21^. Previous in vivo and in vitro studies revealed significant overexpression of MIF in cutaneous melanocytic tumors^19,22,23^. Using *Mif*^*−/−*^ mice, a study by Martin et al*.*^13^ indicated that MIF also plays an important role in the development and progression of UVB-induced NMSC. Our previous study revealed an enhanced expression of MIF in lesional skin of patients with actinic keratosis or cutaneous squamous cell carcinoma (SCC), together pointing towards an important role for MIF in the pathogenesis of NMSC^3^.

Various studies have revealed that D-dopachrome tautomerase (D-DT, also known as MIF-2) is a functional homolog of MIF, since both have a similar gene structure, 3D architecture, and enzyme activity^11,24–26^. D-DT shares several biological activities with MIF and could therefore represent an endogenous amplifier of MIF action^21,27,28^; however, divergent effects have also been reported^28,29^. Nevertheless, little is known about the biological function of D-DT and its role in photocarcinogenesis^30^. A previous study showed increased expression of D-DT in the epidermis of human skin after UVB exposure, similar to the expression of MIF, suggesting that D-DT may play a similar role as MIF in such inflammatory processes^31^. Recently, it was shown that chronic UVB exposure accelerates tumor development in *D-dt*-overexpressing transgenic mice, highlighting D-DT as a functionally important cytokine in photocarcinogenesis^32^.

The present study aimed to assess the pathophysiological function of MIF and its homolog D-DT in human cutaneous inflammatory reactions and the development and progression of UVB- and chemically induced NMSC.

## Results

### Significantly reduced inflammatory response ***in Mif***^*−/−*^, ***D-dt***^*−/−*^ and ***Mif***^*−/−*^***/D-dt***^*−/−*^ mice after acute UVB exposure

**Figure 1:**
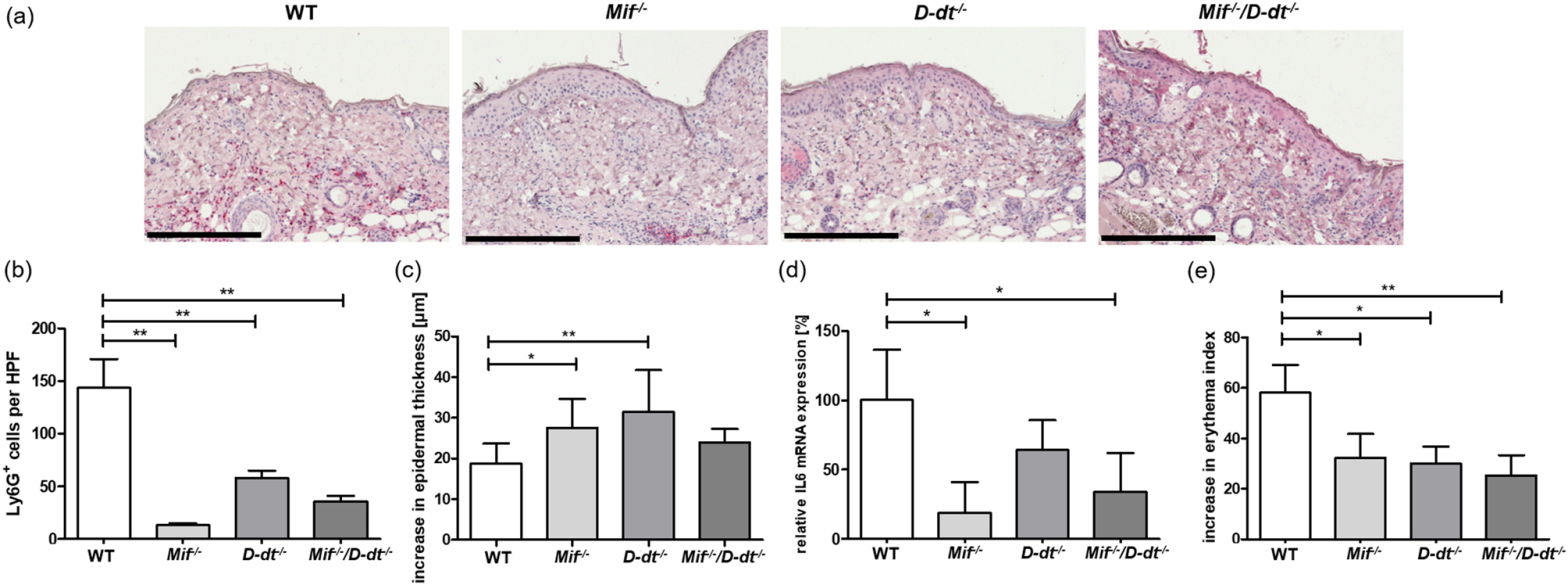
*Mif*^*-*^ and *D-dt* deficient mice exhibit a significant decrease in inflammation after acute UVB exposure. All mice were dorsally irradiated with a single dose of UVB (2240 J/m^2^) and dorsal skin was harvested 48 h later. (**a**) Representative Ly6G-stained sections of dorsal skin samples from UVB-irradiated mice (n = 6 mice per group). (**b**) Quantitative analysis of infiltrating neutrophils in UVB-exposed mice. Ly6G-expressing neutrophils were counted as cells per × 200 magnification, given as cells per high-power field (HPF) (**c**) Increase in epidermal thickness between UVB-irradiated mice versus non-irradiated mice. For each mouse epidermal thickness was measured at six different positions. Data represent mean ± SD. *p < 0.05; **p < 0.01. Scale bars = 200 µm. (**d**) qRT-PCR analysis of IL-6 expression in UVB-irradiated mice. (**e**) Increase in erythema index. Skin color for erythema assessment was measured before and 48 h after UVB irradiation.

To investigate the effects of MIF and D-DT during the acute inflammatory phase, we irradiated WT, *Mif*^*−/−*^, *D-dt*^*−/−*^ and *Mif*^*−/−*^*/D-dt*^*−/−*^ mice dorsally with a single UVB dose of 2240 J/m^2^. We determined the infiltration of Ly6G-positive neutrophils, as this is the hallmark of the acute inflammatory reaction (Fig. 1a). UVB-irradiated *Mif*^*−/−*^*, D-dt*^*−/−*^ and *Mif*^*−/−*^*/D-dt*^*−/−*^ mice exhibited significantly fewer infiltrating neutrophils than WT mice after 48 h (Fig. 1b). In addition, we measured the epidermal thickness of all mice and found that the epidermal thickness was significantly increased in UVB-irradiated *Mif*^*−/−*^ and *D-dt*^*−/−*^ mice compared to WT controls (Fig. 1c). *Mif*^*−/−*^*/D-dt*^*−/−*^ mice also exhibited an increased epidermal thickness, but it was not significantly different when compared to the *Mif*^*−/−*^ and *D-dt*^*−/−*^ strains.

The inflammatory cytokine interleukin (IL)-6 is a known marker of UVB-induced inflammatory reactions^32^. IL-6 expression was significantly reduced in *Mif*^*−/−*^ and *Mif*^*−/−*^*/D-dt*^*−/−*^ mice and slighty decreased in *D-dt*^*−/−*^ mice after UVB irradiation (Fig. 1d).

To further assess the severity of skin inflammation we measured erythema. Consistent with the lower neutrophil infiltration, all KO mice developed significantly less erythema compared to the WT controls (Fig. 1e).

### Delayed and suppressed tumorigenesis in ***Mif***^*−/−*^, ***D-dt***^*−/−*^ and ***Mif***^*−/−*^***/D-dt***^*−/−*^ mice upon chronic UVB irradiation

**Figure 2:**
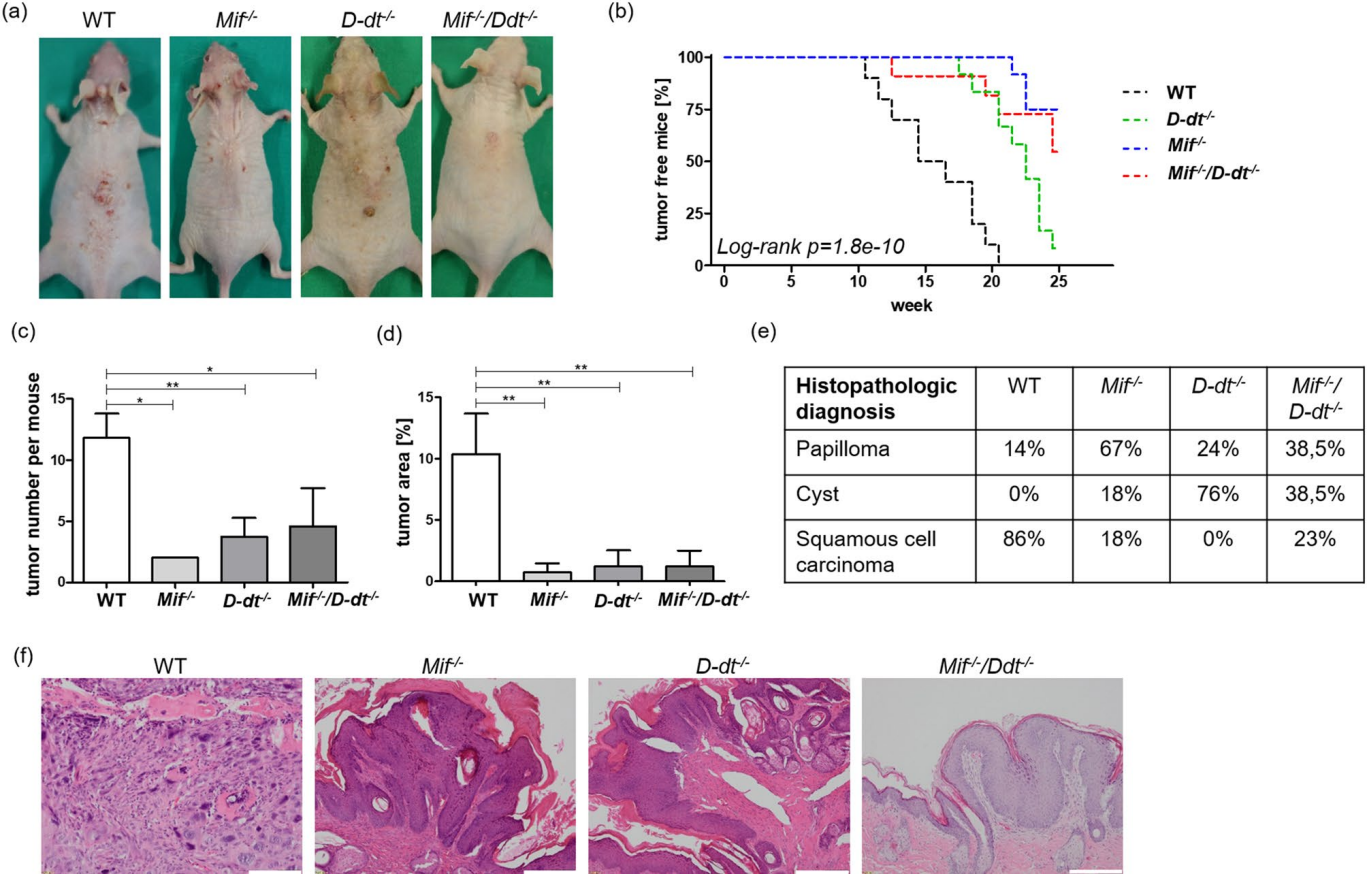
*Mif*^*−/−*^, *D-dt*^*−/−*^ and *Mif*^*−/−*^*/D-dt*^*−/−*^ mice develop significantly later and fewer tumors during chronic UVB irradiation. Mice (n = 12 per group) were irradiated three times weekly for 25 weeks. (**a**) Representative photos of mice from each group. Photos were taken after euthanasia. (**b**) Kaplan–Meier plot showing the percentage of tumor-free mice. (**c**) Number of tumors per mouse in week 25. (**d**) Shown is the percentage of tumor area compared to the total dorsal area of each mouse. (**e**) Histologic classification of epithelial lesions. The incidence of SCC and papillomas in all KO groups was significantly different compared to WT mice (*p* < 0.001). (**f**) Representative H&E stainings of classified lesions in each group. While WT mice displayed less differentiated tumors, the KO mice displayed papillomas. Data represent mean ± SD. *p < 0.05; **p < 0.01. Scale bars = 50 µm.

*Mif*^*−/−*^, *D-dt*^*−/−*^, *Mif*^*−/−*^*/D-dt*^*−/−*^ and WT SKH1 mice developed skin tumors after irradiation with UVB three times a week for 25 weeks (Fig. 2a). While the first tumor of at least 1 mm^2^ occurred in WT mice at week 12, the first *Mif*^*−/−*^*/D-dt*^*−/−*^ mice developed a tumor at week 13, followed by *D-dt*^*−/−*^ mice at week 18 and *Mif*^*−/−*^ mice at week 22 (Fig. 2b). Only the WT group reached a tumor incidence of 100% by week 21. At the end of week 25, only 35% of *Mif*^*−/−*^ mice and 45% of *Mif*^*−/−*^*/D-dt*^*−/−*^ mice developed tumors. On the other hand, 91% of the *D-dt*^*−/−*^ mice had tumors.

Including only mice in which tumors occurred, tumor number was significantly reduced in *Mif*^*−/−*^, *D-dt*^*−/−*^ and *Mif*^*−/−*^*/D-dt*^*−/−*^ mice at week 25 compared to WT controls (Fig. 2c). Consistently, all knockout groups displayed a significantly decreased tumor area per mouse compared to WT controls (Fig. 2d).

In addition, two randomly selected tumors from each mouse were assessed in blinded fashion by a dermatopathologist (Fig. 2e,f). While most tumors of WT mice were classified as SCC, those from each of the three KO mouse strains were predominantly papillomas.

### ***Mif*** and ***D-dt*** deficiency does not influence tumor onset or the number of tumors, but leads to smaller tumors in a murine chemical skin carcinogenesis model

**Figure 3:**
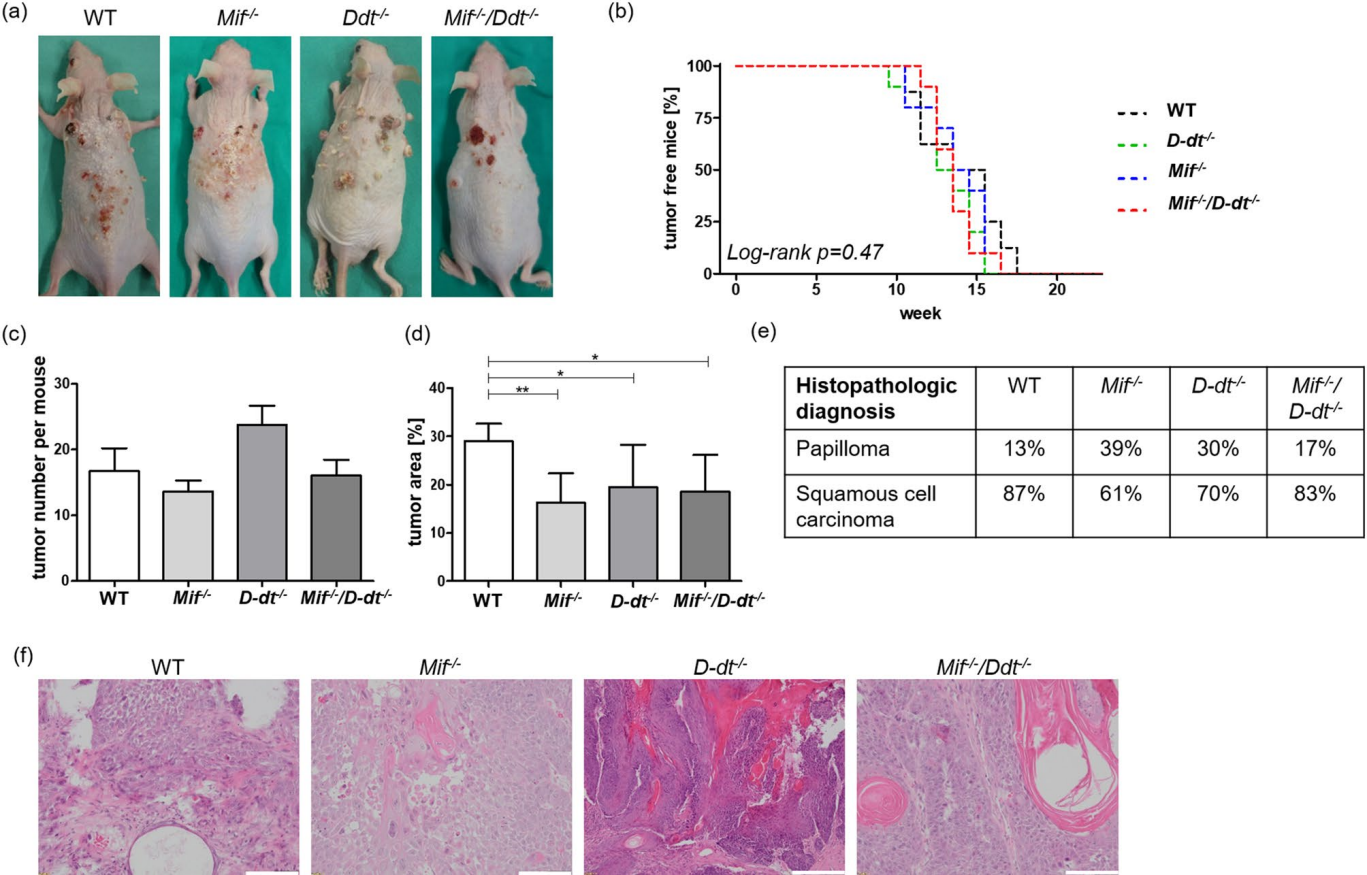
*Mif*^*−/−*^, *D-dt*^*−/−*^ and *Mif*^*−/−*^*/D-dt*^*−/−*^ mice show a reduced tumor area but no differences in the timing of tumor onset and number of tumors after chronic B(α)P treatment. Mice (n = 10 per group) were treated with B(α)P two times weekly for 23 weeks. (**a**) Representative photos of mice from each group. Photos were taken after euthanasia. (**b**) Kaplan–Meier plot showing the percentage of tumor-free mice. (**c**) Number of tumors per mouse in week 23. (**d**) Shown is the percentage of tumor area compared to the total dorsal area of each mouse. (**e**) Histologic classification of epithelial lesions. (**f**) Representative H&E stainings of squamous cell carcinomas in each group. Data represent mean ± SD. *p < 0.05; **p < 0.01. Scale bars = 50 µm.

All mice displayed skin tumors after treatment with B(α)P twice weekly for 23 weeks (Fig. 3a). In contrast to our long-term UVB experiments, we did not observe any differences in the timing of tumor onset between each of the groups (Fig. 3b). First tumors of 1 mm^2^ or larger appeared in each group at week 10 and all groups reached a tumor incidence of 100% at week 17. Consistent with these findings, the number of tumors induced by B(α)P was not statistically different between all groups in week 23 (Fig. 3c). However, *Mif*^*−/−*^, *D-dt*^*−/−*^ and *Mif*^*−/−*^*/D-dt*^*−/−*^ mice showed a significant reduction in the cumulatively measured tumor area, which was comparable to the results of our long-term UVB experiment (Fig. 3d).

Two randomly selected tumors from each mouse were assesed in blinded fashion by a dermatohistopathologist (Fig. 3e,f). The morphology of SCC in all groups appeared identical.

### MIF and D-DT attract macrophages in the in vitro 3D skin model

**Figure 4:**
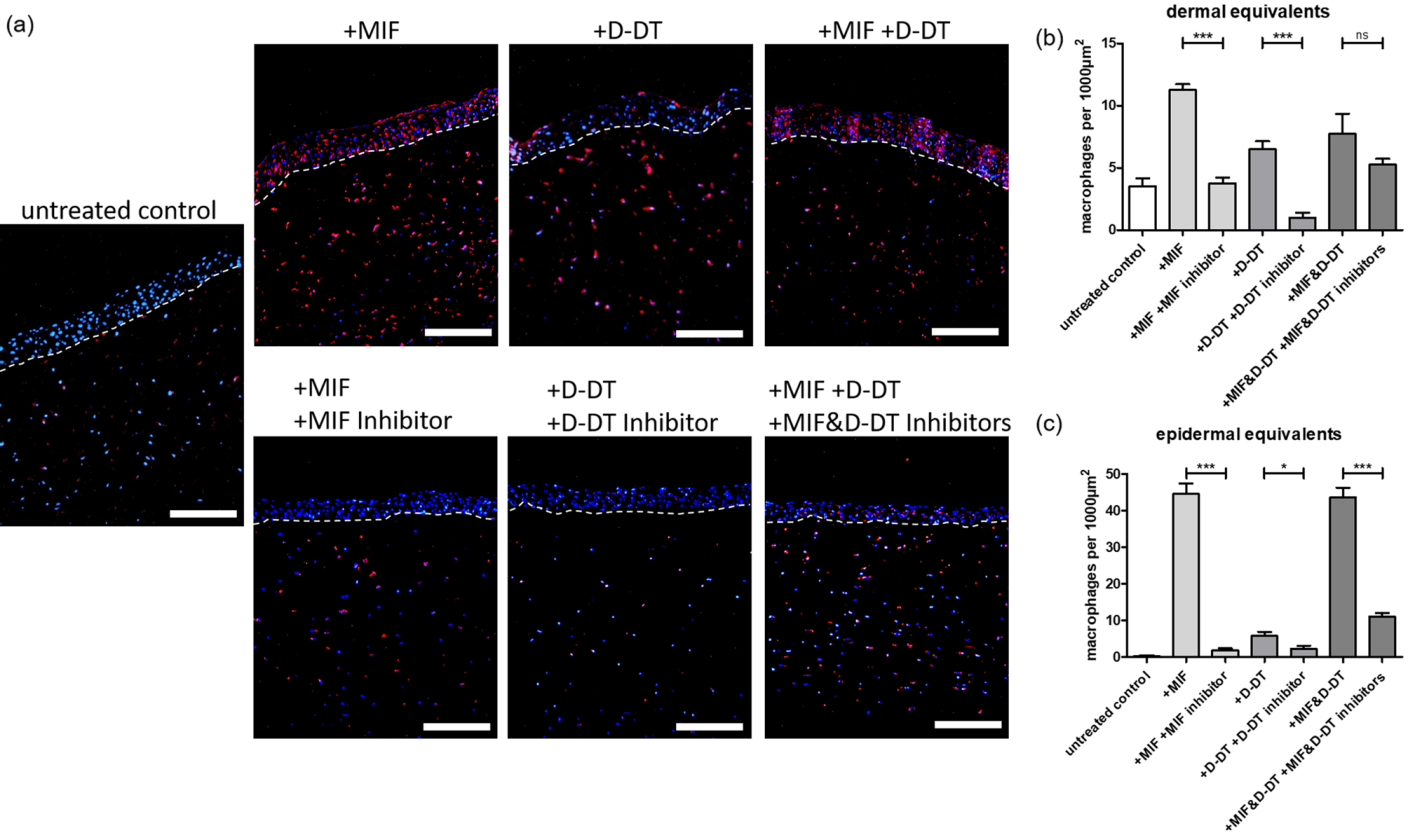
MIF and D-DT provide chemotactic effects on macrophages in an in vitro 3D skin model. 3D skin models containing macrophages were topically stimulated with 30 µl of either human recombinant MIF (10 µg/ml), human recombinant D-DT (10 µg/ml), MIF inhibitor msR4M-L1 (1.3 µM) or D-DT inhibitor 4-CPPC (320 nM). All 3D skin models were harvested 48 h after stimulation. (**a**) Immunofluorescence examination of CD68 in skin models containing macrophages and stimulated by the indicated cytokines and inhibitors. Untreated models served as control. Counterstaining was done with DAPI. (**b**) Quantification of CD68^+^ macrophages per 100 µm^2^ within the dermal equivalents, measured at six representative dermal positions per image. (**c**) Quantification of macrophages per 100 µm^2^ within the epidermal equivalents, measured at six representative epidermal positions per image. Experiments were performed twice independently. Data represent mean ± SD. *p < 0.05; **p < 0.01; ***p < 0.001. Scale bar = 100 µm.

As 3D skin models have proven to be a suitable tool to study the migration of macrophages through tissue^33^, we used such models and incorporated macrophages into the dermal equivalents to study the chemotactic effects of MIF and D-DT, respectively (Fig. 4a). Once the epidermal layer of the full-thickness skin models was stratified, recombinant human MIF, recombinant human D-DT, or both chemokines together were topically applied on the epidermal surface of the models. To further verify the specificity of the recruitment effects, additional models were treated topically with selective inhibitors of MIF (msR4M-L1) or D-DT (4-CPPC). 4-CPPC exhibits a 13-fold selectivity against D-DT over MIF^34,35^ and msR4M-L1 has a fivefold higher affinity for MIF compared to D-DT^36^, and also is specific for MIF interactions with CXCR4^37^, a MIF recruitment receptor prominently expressed on macrophages.

Dermal skin from models was harvested 48 h after stimulation with the cytokines. In untreated control models the incorporated macrophages nearly disappeared (Fig. 4a). In contrast, stimulation with recombinant MIF, D-DT, and the combined treatment stopped the emigration of the macrophages from the models; macrophages even migrated in large numbers to the epidermal layers towards the stimulus of the topically applied cytokines (Fig. 4a upper row). These effects were reversed by additional treatment with the respective inhibitors, with only a small number of macrophages detectable in the dermis and almost no macrophages within the epidermal layers (Fig. 4a lower row). The immunohistochemical observations were confirmed by quantitative measurements of CD68^+^ macrophages in the dermal and epidermal equivalents of the models (Fig. 4b,c).

## Discussion

The most important environmental factor leading to skin cancer is UV radiation, which is estimated to be responsible for almost 90% of NMSCs^38,39^. UV exposure triggers acute inflammation in the skin by stimulating the production of proinflammatory cytokines, including MIF and its homolog D-DT^31,40,41^. The release of such cytokines (as well as neurotransmitters, endocrine factors, and neuropeptides) is a local effect after UVB exposure that can cause systemic effects leading to inflammatory diseases and malignancies^42,43^.

Interesting but sometimes contradictory findings were made in previous studies on the roles of MIF and D-DT in skin tumors. Using *Mif*^−*/*−^ BALB/c mice, Martin and colleagues^13^ showed that MIF has tumor-promoting effects in chronic UVB-induced NMSC. Similar tumor-promoting characteristics were observed for D-DT when Yoshihisa et al*.*^32^ showed that chronic UVB exposure accelerates tumor development in D-DT-overexpressing mice. Our group surprisingly classified MIF as a functional tumor suppressor in chemically-induced skin cancer models^10^. To our knowledge, we now present the first study to simultaneously investigate the role of MIF and D-DT in UVB- and chemically-induced NMSC by developing *Mif*^*−/−*^*, D-dt*^*−/−*^ and *Mif*^*−/−*^*/D-dt*^*−/−*^ mice on a hairless SKH1 background.

In our study, *Mif*^*−/−*^, *D-dt*^*−/−*^ and *Mif*^*−/−*^*/D-dt*^*−/−*^ mice exhibit a significantly reduced inflammatory response after acute UVB exposure as evidences by a reduction in neutrophil infiltration and erythema. These data confirm the previously reported inflammatory effects of MIF and D-DT during the acute response elicited in skin after UVB exposure^13,32^. Although a former study showed additive effects of MIF and D-DT on neutrophil recruitment to the lung^44^, we found that the deletion of only one of the two cytokines appears sufficient to reduce the inflammatory response in skin. In addition to tissue-specific reasons (lung *versus* skin), a mechanistic explanation for this observed discrepancy could be related to differences in cytokine administration/deletion. In the lung study, recombinant MIF or D-DT were locally and transiently administered, whereas in our current study, the global genetic deletion of *Mif* and *D-dt* may elicit secondary compensatory mechanisms.

IL-6 is a key player in inflammatory responses and it is associated with carcinogenesis^45^. Both MIF and D-DT have been shown to induce the expression of IL-6^46,47^. Consistently, the expression level of IL-6 mRNA was decreased in our KO mice after acute UVB exposure, reflecting the lower degree of inflammation in these mice. These observations may be related to the later onset and lower number of tumors in the KO mouse groups observed in our long-term UVB experiments.

Interestingly, in agreement with the data of Martin et al*.*^13^, we observed an increase of epidermal thickness in our KO mice after acute UVB irradiation. In contrast, Yoshihisa et al*.*^32^ showed that D-DT overexpression in transgenic mice significantly increased epidermal thickness after acute UVB exposure. The authors further concluded that a D-DT-dependent increase in cell proliferation could be mediated via activation of the Akt signaling pathway^32^. The results of Martin et al*.* and our study differ from those observations and future studies will be required to clarify the underlying mechanisms. However, it remains speculative that the increase in epidermal thickness plays a protective role and is a reason why the KO mice developed a lower tumor burden during long-term UVB exposure.

There is broad consensus that MIF and D-DT often share similar roles to promote malignant transformation, tumor growth, and metastasis^30^. In our long-term UVB experiment, we found tumor-promoting effects for both cytokines that were consistent with former studies ^13,32,48^. Only a small number of KO animals had developed an SCC compared to the WTcontrols. Putative additive effects of the DKO were not seen. A likely explanation is that MIF and D-DT are both involved in the same pathways. In fact, both cytokines can signal via CD74, the cognate MIF family receptor with key roles in tumorigenesis^49,50^, and recent preliminary evidence also hints at a role for CXCR4 as a shared receptor for MIF and D-DT^9,36^. Nevertheless, these results show that both cytokines are equally important in photocarcinogenesis and may be considered as potential therapeutic targets.

It is surprising that our previous study of chemically-induced skin carcinogenesis in mice of the 129Sv/IMJ or C57Bl/6 backgrounds showed tumor-suppressive effects of *Mif*^10^. Using SKH1 mice in our present B(a)P-induced chemical carcinogenesis model, we found neither tumor-promoting nor tumor-suppressive effects of both cytokines, except that the KO animals showed a smaller tumor area. However, these results are at least consistent with the fact that neither cytokine has a tumor-promoting effect in non-UVB-induced skin cancer. Differences in immune cell recruitment between UVB- and chemically induced skin carcinogenesis could provide an explanation. UVB irradiation leads to multiple immunosuppressive effects that promote the formation of skin cancer^51^. In contrast, in chemical carcinogenesis, a significantly increased infiltration of leukocytes into the skin was observed^52^, which is likely to have an immunostimulatory effect. The fact that we did not observe tumor-suppressive effects of MIF in chemically induced NMSC could be explained by the choice of our mouse strain. It is likely that the SKH1 mouse strain may influence the outcome in our current chemically-induced skin cancer model, as we used only furry mice in our previous study. Although the mutation in the *Hairless* gene increases the susceptibility to UVB-induced tumorigenesis^53^, little is known about the use of SKH1 mice in chemically induced tumorigenesis models. Nevertheless, a few studies already proved that SKH1 mice are sensitive to chemically-induced skin cancer^54^. Thomas et al*.*^55^ showed that SKH1 mice were highly prone to skin carcinogenesis with 7,12-dimethylbenz(a)anthracene (DMBA). The authors discussed that the lack of active hair follicles and the presence of abnormal hair follicles in these mice could indeed reduce the susceptibility to chemicals, but chemicals can still initiate carcinogenesis from the interfollicular epidermis or the rudimentary pilosebaceous appendages in these mice^55^. However, in addition to these anatomical features, SKH1 mice may also exhibit unknown genetic/molecular features that could explain our divergent results.

To get additional insight into the contributing mechanisms, we investigated the chemotactic effects of MIF and D-DT using an in vitro 3D skin model with incorporated macrophages. Topical application of MIF and D-DT to this model led to an accumulation of macrophages in the dermis and especially epidermis that could be reversed by selective MIF and D-DT inhibitors. The MIF inhibitor msR4M-L1 also bears specificity for the MIF/CXCR4 axis. Our findings are in line with studies showing that MIF and D-DT can induce the migration of monocytes and macrophages^9,56,57^. Although previous work has shown additive effects of MIF and D-DT on neutrophil recruitment^44^, our data do not support such additive chemotactic effects. This may be due to the notion that MIF-elicited migration of macrophages can also be mediated by CXCR2, whereas migratory responses induced by both MIF proteins involve interactions with CXCR4^9,28,36,37^, a receptor prominently expressed on both neutrophils and macrophages^58,59^. Nevertheless, our findings substantiate our previously postulated concept that MIF recruits cells of the innate immune system to the skin^10^. Our new data show this effect for the first time also for the MIF family member D-DT. Further investigations, especially on the topical application of MIF and D-DT inhibitors, also in combination with classic UV light protection preparations, would be useful for future in vitro and in vivo tests and perhaps also for later clinical use.

Taken together, we present the first study to investigate the role of MIF and its homolog D-DT in UVB- and chemically-induced NMSC using hairless *Mif*^*−/−*^, *D-dt*^*−/−*^ and *Mif*^*−/−*^*/D-dt*^*−/−*^ mice on a SKH1 background. Our data show that both cytokines have similar inflammatory effects after acute UVB exposure and tumor-promoting effects during chronic UVB irradiation. Observing no additive effects in DKO mice confirms that both cytokines activate a similar signaling threshold by the same receptor pathways. Interestingly, MIF and D-DT do not appear to have a major effect on chemically-induced skin tumors, which may be due to increased tumor immunity in chemical carcinogenesis. This indicates that both cytokines have only a limited tumor-promoting effect that can be neutralized by the immune system. Our data support the assumption that MIF and D-DT are both potential therapeutic targets for the prevention of photocarcinogenesis but not chemical carcinogenesis.

## Materials and methods

### Animals

*Ddt*^*−/−*^*, Mif*^*−/−*^ and *Mif*^*−/−*^*/Ddt*^*−/−*^ mice were recently described^28^. All mice were outbred to the SKH-1 background (Crl:SKH1-Hrhr; Charles River, Sulzfeld, Germany) for 10 generations. For all studies, 8–10 week old male mice were used. At the end of each experiment, mice were euthanized by cervical dislocation. Experimental procedures were approved by the administration of the North Rhine-Westphalian Agency for Nature, Environment and Consumer Protection (Landesamt für Umwelt, Natur und Verbraucherschutz—LANUV, Recklinghausen, Germany—AZ 84-02.04. 2017.A303) and were in accordance with ARRIVE guidelines and the Federation of European Laboratory Animal Science Associations/Society of Laboratory Animal Science (FELASA/GV-SOLAS) Guidelines.

### Acute UVB exposure

Mice were dorsally exposed to one single dose of 2240 J/m^2^ in a UVB research unit (Daavlin, Bryan, Ohio, USA). This dose is the minimal erythemal dose (MED) in SKH-1 mice^54^. Each UVB-irradiated treatment group consisted of 6 mice (n = 6), while non-irradiated control groups consisted of five mice (n = 5). Skin color for erythema assessment was measured with a Mexameter MX18 (Courage + Khazaka electronic GmbH, Cologne, Germany) immediately before UVB irradiation and after 48 h. The measurement is based on absorption/reflection. For the erythema measurement two specific wavelengths are used (green: 568 nm and red: 660 nm), corresponding to the spectral absorption peak of haemoglobin and to avoid other colour influences. The highly sensitive measurement gives values on a broad scale for erythema (0–999). All measurements were conducted in triplicates on the same skin area.

### Chronic UVB exposure

Each treatment group consisted of twelve mice (n = 12), while the corresponding non-irradiated control groups consisted of ten mice (n = 10). Mice were irradiated three times a week (Mondays, Wednesdays, Fridays) for 25 weeks. Mice were irradiated with 2240 J/m^2^ UVB light on treatment days until week 13. During week 14, UVB dosage was increased 10%, with subsequent 10% increases every four weeks until a maximum dosage of 2981 J/m^2^ was reached in week 22. This increase in UVB dose was conducted to account for the adaptation of the skin to UVB irradiation. Tumors > 1 mm^2^ were recorded over time. Percentage of tumor area was determined by comparing tumor area to total dorsal area of each mouse using ImageJ 1.44p software (National Institutes of Health, Bethesda, MD, USA).

### Chemically induced carcinogenesis with B(α)P

Mice (n = 10) were treated dorsally with 100 µg B(α)P (Sigma-Aldrich; solved in 100 µl aceton) twice a week (Mondays and Thursdays) for 23 weeks. Tumors and tumor area were determined as described above.

### Histology, immunohistochemistry and immunofluorescence

Mouse skin tumors fixed in formalin and embedded in paraffin were stained with haematoxylin and eosin (H&E) staining. Moreover, cryosections of the mouse skin were stained for neutrophils with a Ly-6G antibody (553,125 BD, Franklin Lakes, NJ, USA). Macrophages in 4-μm cryosections of 3D skin models embedded in Tissue-Tek O.C.T. ™ (Sakura Finetek) were stained with a CD68 antibody (ab955, Abcam, Cambridge, UK). Photographic documentation was performed using a DMIL microscope (Leica, Wetzlar, Germany).

### RNA isolation and quantitative real-time PCR analysis

RNA was isolated using the high-pure RNA isolation kit (Roche, Mannheim, Germany). RNA yield and purity were measured using a NanoDrop (Thermo, Erlangen, Germany). Purified RNA was reverse-transcribed using TaqMan Reverse Transcription Reagents (Applied Biosystems, Weiterstadt, Germany). TaqMan Gene Expression assays (Applied Biosystems) were used to study the quantitative expression of *IL-6* (Hs00985641_m1) and *Hprt* (hypoxanthine phosphoribosyltransferase; Hs99999909_m1). *Hprt* was used as an internal reference to normalize the target transcript. All measurements were performed in triplicate. The qRT-PCR analyses were executed on an ABI PRISM 7000 Sequence Detection System (Applied Biosystems).

### 3D skin models

Collagen-based 3D skin models comprising NHEK (normal human epidermal keratinocytes) and NHDF (normal human dermal fibroblasts) cells were generated as previously described in a slightly modified version^60^. Macrophages were isolated from peripheral blood mononuclear cells (PBMCs) by adherence two days prior to use for the models. For model development, bovine collagen gels were prepared with NHDF (2 × 10^5^) and macrophages (1 × 10^6^) polymerized in 6-well inserts. The next day, NHEK (2 × 10^6^) were seeded on the gel surface. Following day, 3D skin models were lifted to the air–liquid interface and topically stimulated with 30 µl of either human recombinant MIF (10 µg/ml; Biomol, Hamburg, Germany), human recombinant D-DT (10 µg/ml; Novusbio, Littleton, USA), the MIF peptide inhibitor msR4M-L1 (1.3 µM)^37^ or D-DT inhibitor 4-CPPC (320 nM)^34^.

### Statistical analysis

Statistical analysis was performed using GraphPad PRISM version 7 (La Jolla, CA, USA). Values of *p < 0.05, **p < 0.01 and ***p < 0.001 were considered significant. Mann–Whitney U test was used to compare two groups. Percentage of tumor free mice was calculated and analyzed using the log-rank test. Comparison of tumor incidence was calaculated with the Χ^2^ test.

## Data Availability

This paper does not include large-scale databases (next-generation sequencing or microarray). However, all data generated during and/ or analysed during these studies are available from the corresponding author on reasonable request.
